# How do schools influence the emotional and behavioural health of their pupils? A multi-level analysis of 135 schools in the Born in Bradford inner city multi-ethnic birth cohort

**DOI:** 10.1007/s00127-023-02608-8

**Published:** 2024-01-09

**Authors:** Dan Lewer, Simon Gilbody, Gemma Lewis, Joseph Pryce, Gillian Santorelli, Ruth Wadman, Aidan Watmuff, John Wright

**Affiliations:** 1 Bradford Institute for Health Research, Bradford Royal Infirmary, Duckworth Lane, Bradford, BD9 6RJ UK; 2 Department for Epidemiology and Public Health, University College London, 1-19 Torrington Place, London, WC1E 7HB UK; 3 Department of Health Sciences, University of York, York, YO10 5DD UK; 4 Division of Psychiatry, University College London, 149 Tottenham Court Road, London, W1T 7NF UK; 5 Hull York Medical School, University of York, York, YO10 5DD UK

**Keywords:** Schools, Mental health, Child behavior, School health services

## Abstract

**Purpose:**

To estimate variation in emotional and behavioural problems between primary schools in Bradford, an ethnically diverse and relatively deprived city in the UK.

**Methods:**

We did a cross-sectional analysis of data collected from 2017 to 2021 as part of the ‘Born In Bradford’ birth cohort study. We used multilevel linear regression in which the dependent variable was the Strengths and Difficulties Questionnaire (SDQ) total score, with a random intercept for schools. We adjusted for pupil-level characteristics including age, ethnicity, socioeconomic status, and parental mental health.

**Results:**

The study included 5,036 participants from 135 schools. Participants were aged 7–11 years and 56% were of Pakistani heritage. The mean SDQ score was 8.84 out of a maximum 40. We estimated that the standard deviation in school-level scores was 1.41 (95% CI 1.11–1.74) and 5.49% (95% CI 3.19–9.37%) of variation was explained at school level. After adjusting for pupil characteristics, the standard deviation of school-level scores was 1.04 (95% CI 0.76–1.32) and 3.51% (95% CI 1.75–6.18%) of variation was explained at school level. Simulation suggested that a primary school with 396 pupils at the middle of the distribution has 63 pupils (95% CI 49–78) with a ‘raised’ SDQ score of 15 + /40; and shifting a school from the lower to the upper quartile would prevent 26 cases (95% CI 5–46).

**Conclusion:**

The prevalence of emotional and behavioural problems varies between schools. This is partially explained by pupil characteristics; though residual variation in adjusted scores may suggest that schools have a differential impact on mental wellbeing.

**Supplementary Information:**

The online version contains supplementary material available at 10.1007/s00127-023-02608-8.

## Introduction

Schools are central to children’s lives, and may have an important effect on emotional and mental wellbeing. Proposed mechanisms through which schools might affect mental health include learning of prosocial behaviours from school activities that involve positions of responsibility; building of trust and reciprocity in relationships between staff and students, which encourages beneficial relationships both within closely bonded groups (e.g., friendship groups) and with more distant people (e.g., between teachers and pupils); and the school’s ability to support pupils’ autonomy and cultural alignment with the school, which can be undermined by a narrow focus on academic attainment [1–3]. All of these theories include the concept that schools can support emotional and mental wellbeing by encouraging institutional participation and prosocial roles, and where this is not successful pupils might seek alternative types of belonging and peer validation, which may involve substance use, bullying violence, or other unhealthy behaviours. Negative experiences at school are common—for example, one-in-five ten-year-olds in England and Wales report being bullied in the past 12 months [4], and in a survey of young people in the UK, “pressure to do well at school or college” was the most common factor affecting mental health [5].

Building on these theories, many school-based interventions aiming to improve pupil’s mental health and wellbeing have been developed, and there is a large body of research evaluating these interventions. For example, a review of school-based depression and anxiety prevention programs included 142 trials [6], while a review of mindfulness-based programmes included 66 trials [7]. Most of these trials are small with methodological issues [8, 9], though some larger trials of ‘whole-school’ approaches have found benefits in terms of outcomes such as bullying and pupil’s feelings about the school [10–12]. In some cases, evidence appears conflicting, such as the null finding from a recent large trial of school-based mindfulness [13], which contrasted with previous systematic reviews of smaller studies that suggested small but positive effects [7]. Furthermore, many schools implement mental health-related interventions that do not have a clear evidence base. In the UK, for example, an intervention called ‘Mental Health Support Teams’ is being rolled out nationally despite a lack of quantitative evidence [14]. The landscape of school-based mental health interventions is complex, with a large number of competing interventions and extensive but unclear evidence in many areas.

Schools serve different communities, and, therefore, the composition of the student body varies in ways that is associated with mental health and wellbeing. For example, low socioeconomic status is strongly associated with emotional and behavioural problems[15] and higher rates of mental health problems [16]. Among state-funded primary schools in the UK, the proportion of children who are eligible for free school meals (a marker of low family income) ranges from zero to more than three-quarters [17]. Family issues including parental mental health problems [18, 19] and other adverse childhood experiences [20] are strongly associated with poor mental health outcomes, and the prevalence of these experiences varies geographically [21]. This causes variation between schools in the mental health of pupils independently of school-level factors such as policies and interventions.

Therefore, the mental health and wellbeing of pupils is likely to vary between schools as a result of both pupil- and school-level factors. Empirical studies using multilevel modelling have found substantial differences between schools in terms of tobacco use, alcohol, drugs, and violence [1]. A smaller number of studies have investigated school-level variation in mental health, finding that between 1 and 4% of variation was explained at school-level after adjustment for pupil characteristics [22–26]. This study aims to test the effect of primary schools within Bradford, UK, on pupils’ mental health. We expected that pupils' mental health would vary substantially between schools, and this variation would be mostly explained by the characteristics of pupils such as socioeconomic status and parental mental health.

## Methods

We did a cross-sectional analysis using data from a community-based cohort of children in Bradford, UK. The analysis followed a pre-registered protocol [27].

### Data source

We used data from Born In Bradford, a birth cohort study of individuals born at a hospital in Bradford between 2007 and 2011 [28]. Bradford is a city in West Yorkshire, England, UK. It has high levels of socioeconomic deprivation and ethnic diversity compared with the rest of the UK. We did a cross-sectional analysis of data from a follow-up of participants at age 7–11. This follow-up is known as ‘Growing Up’ [29]. Growing Up included community-based family assessments done during home visits or in community settings such as children’s centres, schools, or GP practices. Fieldwork was done between 2017 and 2021. Parents completed a self-administered questionnaire on a tablet computer, which included questions about the household structure, social factors, and family health. 12,679 parents and guardians were invited to participate in this survey, and 5,390 participated (43%). We excluded participants who had started secondary school.

For descriptive purposes, we also used school-level variables from the Department for Education’s register of schools[30] and Ofsted (the government organisation that inspects schools in England) [31]. This information included the school headcount, most recent Ofsted result, the school’s funding model, and the proportion of pupils eligible for free school meals.

### Variables

The outcome was the parent-reported Strengths and Difficulties Questionnaire (SDQ), a 25-item questionnaire measuring emotional and behavioural problems [32]. The primary outcome was the total difficulties score, with a maximum score of 40 (the most problems), and secondary outcomes were four subscales measuring emotional problems, conduct problems, hyperactivity, and peer problems, each with a maximum score of 10.

The main exposure was the primary school that children attended at the time of the survey.

**Fig. 1:**
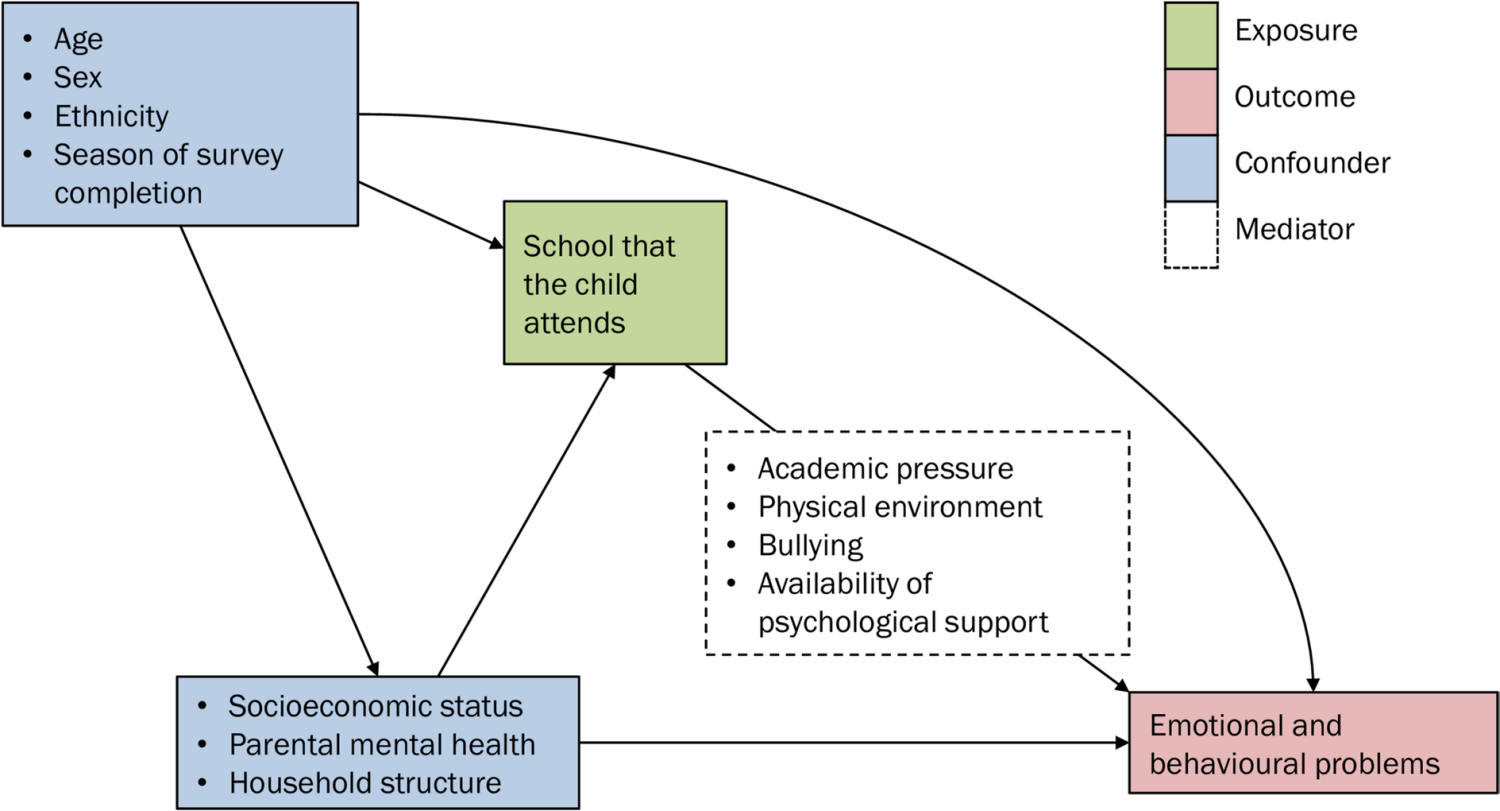
Causal model of the effect of schools on emotional and behavioural problems

We selected potential confounding variables based on an a-priori causal model (Fig. 1). Pupils may differ across schools in terms of these characteristics, and these characteristics may also affect mental health. The confounding variables were age at survey completion; sex; ethnicity, grouped as Pakistani, White, Asian other than Pakistani, Mixed, Other, and Black; the season and weekday when the survey was completed; the highest of the parents’ occupational statuses, measured using the National Statistics Socio-economic classification; the deprivation of the neighbourhood where the participant lives, measured using quintiles of the Index of Multiple Deprivation; the mother's mental health, measured using the 8-item Patient Health Questionnaire depression scale; and the household size.

### Statistical analysis

We described the characteristics of individual participants and schools.

We then used a mixed linear model to estimate school-level effects on mental health. We first estimated a model in which the dependent variable was the SDQ total difficulties score, a random intercept for the school, and no fixed effects. We fit the model using a restricted maximum likelihood method implemented in the R package ‘lme4’ [33]. We used this model to estimate school-level crude scores and 95% confidence intervals. We tested statistical evidence of variation between schools using a likelihood ratio test comparing models with and without random intercepts, with the mixed model re-estimated using maximum likelihood. We reported the standard deviation of random effects and estimated the intraclass correlation (the proportion of variance that is explained at the school level) with bootstrapped confidence intervals.

We then adjusted for pupil characteristics by adding fixed effects for the variables listed above as potential confounders. We included linear and quadratic terms for age, and a linear term for the mother’s PHQ8 score. Other variables were categorical. After adjusting for these potential confounders, we reported the adjusted standard deviation of school-level scores and the adjusted intraclass correlation.

We repeated these procedures for secondary outcomes (the SDQ subscales).

There was some missing data for the index of multiple deprivation (1% of observations), socioeconomic status of parents (5%), and the mother’s PHQ8 score (3%). We used multiple imputation to generate 20 complete datasets, repeated the analysis using each dataset, and combined the results using Rubin’s Rule. For p-values, we used the median *p*-value from imputed datasets. The complete datasets were generated using the R package ‘Amelia’ [34].

**Fig. 2:**
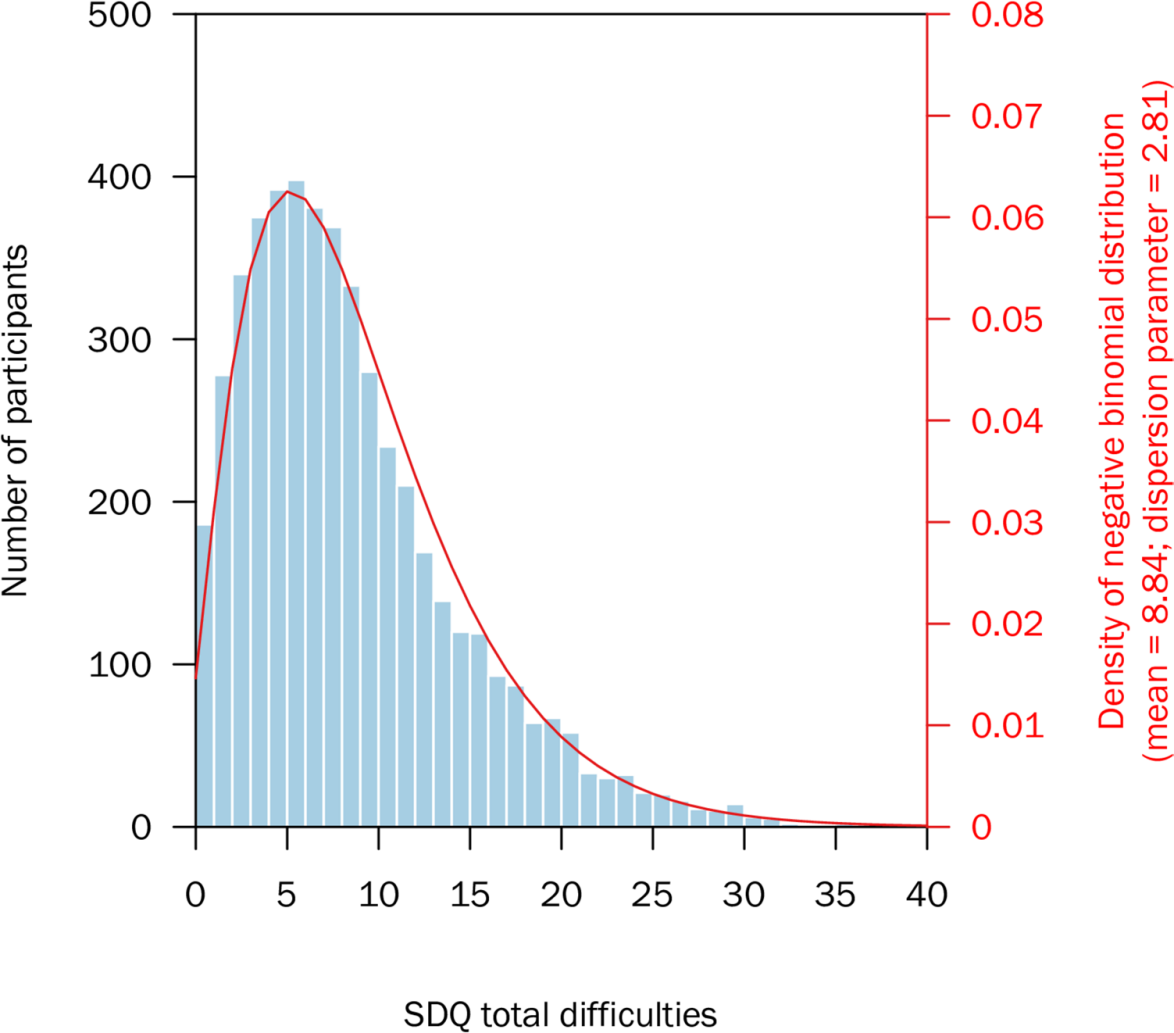
Histogram of SDQ total difficulties scores for 5,036 primary school pupils in Bradford, UK, at age 7–11

Finally, we did a simulation exercise to contextualise the school-level variation. We estimated the number of cases in an average-sized primary school of 396 pupils assuming cutoffs in the SDQ total difficulties scores. We assumed a negative binomial distribution in the SDQ score with a dispersion parameter estimated from the whole dataset (theta = 2.81; see Fig. 2), and mean values drawn from specified quantiles of the distribution of school-level means, estimated from the mixed linear model. We simulated 10,000 schools at the lower quartile, median, and upper quartile of SDQ total difficulties score. The SDQ is intended as a score rather than to identify ‘cases’ using cutoffs, but a score of 15/40 is sometimes considered ‘slightly raised’ while a score of 20/40 is sometimes considered ‘very high’ [35]. Therefore we used cutoffs of 15 and 20 and calculated the number of ‘cases’ in each simulation; then reported the 2.5%, 50%, and 97.5% quantiles.

Analysis was done using R version 4.2.0. The analysis code is available at https://github.com/danlewer/bib_schools.

### Ethics and approvals

Ethical approval for the Born In Bradford ‘Growing Up’ survey was obtained from the National Health Service Health Research Authority Yorkshire and the Humber (Bradford Leeds) Research Ethics Committee for the community-based family assessments and school-based measures (reference: 16/YH/0320) and the school-based cognitive and wellbeing assessments (reference: 16/YH/0062). This analysis was approved by the Born In Bradford executive (reference: SP619).

## Results

### Characteristics of pupils and schools

We studied 5,063 children in 135 primary schools in Bradford. The median age was 9.75 years (IQR 9.00–10.33); 2,461 (49%) were female; 2,828 (56%) had Pakistani ethnicity; and 3,442 (68%) lived in the most deprived quintile of neighbourhoods (when all neighbourhoods in England are ranked). Characteristics of participants are summarized in Table 1.

**Table 1:** Characteristics of participants in the Born In Bradford “Growing Up” survey

Variable	Level	*n* (%)
Total		5,063 (100.00)
Age when survey was completed	7	156 (3.08)
	8	1,088 (21.49)
	9	1,766 (34.88)
	10	1,777 (35.10)
	11	268 (5.29)
	6 or 12	8 (0.16)
	Median [IQR]	9.75 [9.00–10.33]
Sex	Female	2,461 (48.61)
	Male	2,602 (51.39)
Ethnicity	Pakistani	2,828 (55.89)
	White British and White Other	1,393 (27.53)
	Asian (other than Pakistani)	500 (9.88)
	Mixed	210 (4.15)
	Other	68 (1.34)
	Black African and Black Caribbean	61 (1.21)
Index of multiple deprivation, quintile	1 (most deprived)	3,442 (67.98)
	2	1,068 (21.09)
	3	283 (5.59)
	4	155 (3.06)
	5 (least deprived)	55 (1.09)
	Missing	60 (1.19)
Household size	1–3	577 (11.43)
	4–5	2,477 (49.05)
	6–7	1,474 (29.19)
	8 +	522 (10.34)
Socioeconomic status of parent (NS-SEC)	Managerial, administrative and professional occupations	1,723 (34.87)
	Intermediate occupations	795 (16.09)
	Small employers and own account workers	836 (16.92)
	Lower supervisory and technical occupations	481 (9.73)
	Semi-routine and routine occupations	842 (17.04)
	Missing	264 (5.34)
PHQ8 score of mother (/24)*	0–4 (No or minimal symptoms)	3,434 (67.83)
	5–9 (Mild symptoms)	898 (17.74)
	10–14 (Moderate symptoms)	324 (6.40)
	15–24 (Severe symptoms)	259 (5.12)
	Missing	148 (2.92)
	Median [IQR]	2 [0–6]
SDQ total difficulties score (/40)*	0–14 (Close to average or normal)	4,184 (82.64)
	15–17 (Slightly raised)	332 (6.56)
	18–19 (High)	151 (2.98)
	20–40 (Very high)	330 (6.52)
	Missing	66 (1.30)
	Median [IQR]	8 [4–12]

*IQR* interquartile range, *SDQ* strengths and difficulties questionnaire, *PHQ8* Patient Health Questionnaire depression scale, *NS-SEC* National Statistics Socio-economic Classification

*Categories are shown for descriptive purposes and are not used in analysis. PHQ8 categorisation is based on a report from the US Centers for Disease Control and Prevention [36]. SDQ categorisation is based on a report from the UK Office for National Statistics [35]

The 135 primary schools had a total headcount of 54,887, meaning that 9.2% of primary school children in Bradford participated. The median participants per school was 29 (IQR 16–51). Characteristics of schools are summarized in Table 2.

**Table 2:** Characteristics of schools included in the study

Variable			Median [IQR]
			Number (%)
Number of primary schools included in analysis			135
Variables from the Department for Education school census	Headcount		416 [234–468]
	Percent eligible for free school meals		26 [17–33]
	School type	Academies and free schools	69 (51.11)
		Local authority maintained schools	63 (46.67)
		Independent schools	3 (2.22)
	Most recent Ofsted rating	Outstanding	11 (9.73)
		Good	85 (75.22)
		Requires improvement	16 (14.16)
		Special Measures	1 (0.88)
	School gender	Girls only	1 (0.74)
		Mixed	134 (99.26)
	Deprivation quintile of the neighbourhood in which the school is located	5—most deprived	65 (48.15)
		4	28 (20.74)
		3	14 (10.37)
		2	8 (5.93)
		1—least deprived	5 (3.70)
		Missing	15 (11.11)
Variables from the BIB growing up survey	Participants in BIB growing up		29 [16–51]
	Age at participation (years)		9.75 [9.58–9.92]
	School-level mean SDQ score	Total difficulties (/40)	8.81 [8.00–10.01]
		Conduct problems (/10)	1.58 [1.32–1.89]
		Emotional problems (/10)	2.18 [1.90–2.46]
		Hyperactivity (/10)	3.46 [3.07–3.93]
		Peer problems (/10)	1.58 [1.35–1.93]

*IQR* interquartile range, *SDQ* strengths and difficulties questionnaire

The median SDQ total difficulties score was 8 (IQR 4–12) and the mean was 8.84; and pupil-level scores approximated to a negative binomial distribution with dispersion parameter of 2.81 (Fig. 2). Histograms of subscale scores are included in Supplementary Information.

### School-level variation in outcomes

**Fig. 3:**
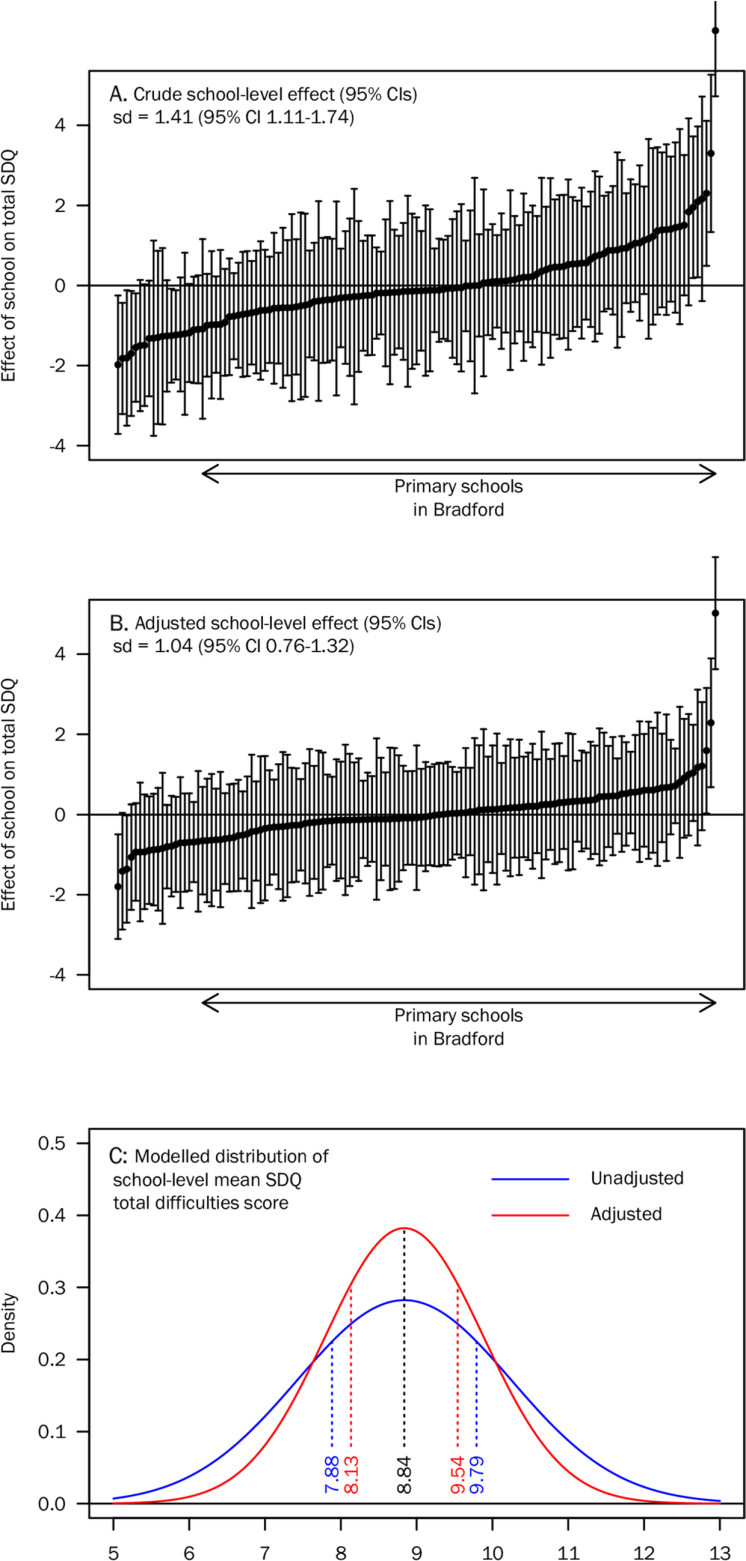
Distribution of school-level SDQ total difficulties scores at primary schools in Bradford. Panels **A** and **B** show the mean SDQ score for each school, estimated using random intercepts from the multilevel linear model. In Panel **B**, school-level means are adjusted for differences in pupil characteristics. Panel **C** shows the distribution of school mean scores based on the estimated standard deviation in random intercepts from the multilevel linear model, with lower and upper quartiles marked

Before adjusting for pupil characteristics, we found strong evidence of variation in the SDQ total difficulties scores between schools (*p* < 0.001). The standard deviation in school-level SDQ scores was 1.41 (95% CI 1.11–1.74), meaning that a school at the lower quartile had a mean SDQ score of 7.88; while a school at the upper quartile had a mean SDQ score of 9.79 (Fig. 3). The intraclass correlation suggested that 5.49% (95% CI 3.19–9.37%) of variation in the crude SDQ total difficulties score was explained at school level.

After adjusting for pupil characteristics, there remained strong evidence of variation in SDQ total difficulties scores between schools (*p* < 0.001). The standard deviation in school-level scores was 1.04 (95% CI 0.75–1.32), meaning that a school at the lower quartile had a mean adjusted score of 8.13; while a school at the upper quartile had a mean adjusted score of 9.54. The intraclass correlation suggested that 3.51% (95% CI 1.75–6.18%) of variation in the SDQ total difficulties score was explained at school level after adjustment for pupil characteristics.

Using a cutoff in the SDQ total difficulties score of 15/40, we estimate that a typical school with 396 pupils would have 63 cases (95% CI 49, 78). A school at the lower quartile of the distribution (i.e., fewer pupils have emotional and behavioural problems) would have 46 cases (95% CI 34, 59), while a school at the upper quartile would have 81 cases (95% CI 66, 97), such that the difference between the lower and upper quartiles is 35 cases (95% CI 15, 55). After adjusting for pupil characteristics, we estimated a difference of 26 cases (95% CI 5, 46) between the lower and upper quartiles. This is an estimate of the impact of school-level variables on the number of children with emotional or behavioural problems. Results using a cutoff in the SDQ total difficulties score of 20/40 are also shown in Table 3.

**Table 3:** Estimated number of ‘cases’ in a primary school with 396 pupils at specified cutoffs of the SDQ total difficulties score, and the difference between schools at the lower and upper quartiles of the distribution (95% CIs)

Position in distribution of schools	SDQ cutoff = 15	SDQ cutoff = 20
Middle	63 (49, 78)	24 (15, 34)
Crude number of cases		
Lower quartile	46 (34, 59)	15 (8, 23)
Upper quartile	81 (66, 97)	34 (24, 45)
Difference	35 (15, 55)	19 (6, 32)
Adjusted number of cases		
Lower quartile	50 (38, 64)	17 (10, 26)
Upper quartile	76 (61, 92)	31 (21, 42)
Difference	26 (5, 46)	14 (1, 27)

Fixed effects from the adjusted model suggest that higher SDQ scores (i.e., more emotional and behavioural problems) are associated with living in a more deprived neighbourhood; White British and White Other, or Mixed ethnicities (in comparison to Pakistani ethnicity); higher PHQ8 scores for the participant’s mother (i.e., greater symptoms of depression); lower status socioeconomic classifications of the participant’s parents; and smaller household size (Table 4). These associations should be treated with caution because the analysis was not designed to estimate causal effects of these variables, and interpreting these coefficients causally is known as the ‘Table 2 fallacy’ [37].

**Table 4:** Results from multilevel model: fixed effects, showing the association between pupil-level characteristics and SDQ total difficulties score

Variable	Level	Adjusted regression coefficient (95% CI)
Age	Linear	−7.86 (−19.05, 3.33)*
	Quadratic	−8.53 (−19.61, 2.54)*
Sex (ref = female)	Male	1.22 (0.91, 1.54)
Season when the survey was completed (ref = winter)	Spring	0.04 (−0.44, 0.51)
	Summer	0.28 (−0.19, 0.74)
	Autumn	0.24 (−0.20, 0.69)
Index of multiple deprivation, quintile (ref = 1, most deprived)	2	−0.46 (−0.89, −0.03)
	3	−1.00 (−1.79, −0.22)
	4	−1.60 (−2.69, −0.51)
	5 (least deprived)	−2.33 (−4.03, −0.63)
	Missing	−0.18 (−1.74, 1.37)
Ethnicity (ref = Pakistani)	White British and White Other	1.69 (1.19, 2.19)
	Asian (other than Pakistani)	−0.19 (−0.76, 0.38)
	Mixed	1.53 (0.68, 2.38)
	Other	−0.61 (−2.06, 0.84)
	Black African and Black Caribbean	−0.43 (−1.93, 1.07)
PHQ8 score of mother		0.38 (0.34, 0.41)
Socioeconomic status of parents (ref = Managerial, administrative and professional)	Intermediate occupations	0.58 (0.08, 1.08)
	Small employers and own account workers	0.24 (−0.26, 0.74)
	Lower supervisory and technical occupations	0.53 (−0.06, 1.13)
	Semi-routine and routine occupations	0.66 (0.16, 1.16)
	Missing	0.33 (−0.45, 1.10)
Household size (ref = 1–3)	4–5	−0.69 (−1.23, −0.14)
	6–7	−0.76 (−1.36, −0.16)
	8 +	−0.81 (−1.55, −0.08)

*CI* confidence interval, *PHQ8* Patient Health Questionnaire depression scale

*Participants’ age in years was included as continuous linear and quadric terms (i.e., age and age squared)

Results for SDQ subscales are shown in Table 5. The results were broadly similar for conduct, hyperactivity and peer problems, with strong evidence of variation across primary schools in the crude and adjusted scores (p < 0.001); and variation in the crude scores being partially explained by pupil characteristics. For emotional problems, the variation between schools appeared smaller, with an intraclass correlation of 0.38% (95% CI 0.01%, 1.80%), and no statistical evidence of variation (p = 0.32 for crude scores and p = 0.55 for adjusted scores).

**Table 5:** School-level variation in subscales of the Strengths and Difficulties Questionnaire

SDQ score	Crude variation		Adjusted variation**	
	Standard deviation of school-level score (95% CI)	Intra-class correlation* (95% CI)	Standard deviation of school-level SDQ (95% CI)	Intra-class correlation* (95% CI)
Total difficulties/40	1.41 (1.11, 1.74)	5.48 (3.19, 9.37)	1.04 (0.76, 1.32)	3.51 (1.75, 6.18)
Conduct problems/10	0.35 (0.26, 0.43)	4.40 (2.06, 7.01)	0.22 (0.13, 0.29)	1.96 (0.54, 3.73)
Emotional problems/10	0.13 (0.00, 0.24)	0.38 (0.01, 1.80)	0.10 (0.00, 0.21)	0.27 (0.00, 1.33)
Hyperactivity/10	0.57 (0.44, 0.70)	4.67 (1.95, 7.42)	0.46 (0.33, 0.57)	3.36 (1.29, 5.83)
Peer problems/10	0.38 (0.30, 0.47)	4.86 (2.56, 7.37)	0.33 (0.25, 0.41)	3.86 (1.85, 6.55)

*SDQ* strengths and difficulties questionnaire, *CI* confidence interval

*Displayed as a percentage (the percentage of total variation explained at school level)

**Adjusted for child’s age; sex; ethnicity; season and weekday of survey completion; parents’ occupation; neighbourhood deprivation; mother's mental health; and household size

## Discussion

### Key findings

Emotional and behavioural problems vary between primary schools. This is partially explained by the characteristics of pupils; though residual variation in adjusted scores may suggest that primary schools have a differential impact on pupils’ mental wellbeing.

### Comparison with other studies

A small number of previous studies have examined the effect of schools on pupils’ mental health. As in the present study, these studies used multilevel analysis of cross-sectional data. In a study of 87,341 pupils at 458 secondary schools in Finland, schools accounted for 1.0% of variation in subjective wellbeing [25]. A study of 23,215 pupils at 648 primary schools in England found that 4.3% of variation in emotional and behavioural problems was accounted for at school level [22]. Three studies of secondary school pupils in England found that 1.4% of variation in mental and emotional health was explained at school level.[23] A study of 26,855 pupils at 85 secondary schools in the UK found that 2.4% of variation in ‘psychopathology’; 1.6% of variation in depression, and 1.4% of variation in wellbeing were explained at school level [24]. All these studies adjusted for pupil characteristics such as socioeconomic status, ethnicity, and age. These intraclass correlations are comparable to the findings from the present study that 3.51% (95% CI 1.75%, 6.18%) of variation in emotional and behavioural problems in primary school children in Bradford, England, was explained at school level. Although intraclass-correlations of less than 10% are sometimes considered low and indicate that most variation is at individual-level [1], these school-level differences can still be important at the population level, as demonstrated in our simulation. There are many more studies of school-level variation in health behaviours such as smoking, diet, and physical activity, and the intraclass correlations in these studies suggest greater variation between schools than for mental health and wellbeing [1].

In this sample, the mean SDQ total difficulties score was 8.84. In a national cross-sectional survey of young people’s mental health [38], the mean score for 6–10 years-olds was 8.0 in 2017, 9.4 in 2020, and 9.8 and 2021. Young people’s mental health appears to have worsened during the COVID-19 pandemic. Direct comparisons between the Born In Bradford and national data are, therefore, difficult without more detailed stratification by time period; however the total SDQ scores in the present study are approximately similar to national data.

### Implications for policy and practice

The variation in emotional and behavioural problems between schools after adjusting for pupil-level factors may suggest that schools have a differential impact on pupils’ mental health. By shifting an average-sized primary school from the lower to the upper quartile of the distribution, we estimated that 26 ‘cases’ would be prevented (95% CI 5–46 cases).

There are effective school-based interventions to promote good mental health and support pupils with mental health problems. Most evidence focuses on selected and indicated interventions, which target people with risk factors or specific mental health problems respectively. For example, trials of school-based cognitive and behavioural therapies for pupils with symptoms of depression and anxiety have found small-to-medium sized effects [39]. A key problem is that these interventions are not scalable, because they rely on trained therapists. They also typically aim to treat rather than prevent mental health problems. Digital interventions may be more scalable, but need better quality evaluation [40].

Whole-school interventions have the potential to improve mental health and prevent mental health problems. There are many plausible mechanisms through which the school environment may affect mental health, including both negative factors such as bullying, violence, and academic pressure; and positive factors such belonging and community, and recognition of success. Trials of whole-school interventions in the UK[10] and India[11, 41] have shown that whole-school interventions can improve pupils’ perceptions of the school environment. In contrast, a recent trial of classroom-based mindfulness in the UK found no meaningful effect on various measures of mental wellbeing [13]. A review of UK-based whole-school interventions to improve mental and emotional well-being identified 12 evaluations with various methodological issues; and small effect sizes from the more robust studies [42]. Overall, there has been limited research into whole-school approaches to improving mental health, despite the scalability.

The results suggest that there is limited variation between primary schools in emotional problems, with intraclass correlation for the emotional subscale of SDQ being 0.38% (95% CI 0.01%, 1.80%), compared to 4–5% for the other subscales. This is not explained by the distribution of pupil-level scores, which was similar for each subscale (shown in Supplementary Information). It may be because parental reports of emotional problems are less accurate than for other subscales; or because schools have a greater effect on behaviour (i.e., peer problems, conduct problems, and hyperactivity) than emotions.

The data presented here also support the large body of evidence that individual pupil characteristics such as socioeconomic status affect mental health. The study was primarily designed to estimate the distribution in school-level effects on mental health, and as such the associations between individual-level variables and SDQ scores presented in Table 4 should be treated with caution. These results suggest that deprivation and poor parental mental health are strongly associated with emotional and behavioural problems, reflecting existing evidence [16, 19]. One potentially surprising association was that children living in larger households appeared to have fewer emotional and behavioural problems. This could be investigated in focused research.

## Strengths and limitations

This study used a widely validated outcomes measure (the Strengths and Difficulties Questionnaire). Compared to previous studies [22–25], this study adds crude measures of school-level variation that allow interpretation of the moderate role of confounding by pupil-level characteristics; and simulation-based estimates of the importance of school-level variation.

The study has three key limitations. First, we could not identify which specific schools have particularly high or low prevalence of emotional and behavioural problems. This was partly because schools in dataset were pseudonymised, meaning we did not have access to their names and locations; and partly because the analysis is designed to estimate the distribution of scores across schools rather scores for individual schools. This approach, therefore, cannot identify specific schools for intervention.

Second, the SDQ questionnaire may be interpreted differently according to the ethnic and language background of the participant and their parents. The SDQ has been validated in many settings, though scores may not be fully comparable between different ethnic groups. For example, a study in the Netherlands found that the factor structure of SDQ was different for children with Dutch and non-Dutch ethnic backgrounds [43]. This may mean that school-level differences are partly explained by ethnic differences in pupils that are not fully described by the variables in our study.

Third, the fixed effects that estimate associations between pupil characteristics and emotional and behavioural problems are difficult to interpret, because the study was not designed to estimate these associations. We have included these results in Table 4 to help readers understand our method and support hypothesis generation in future research.

## Conclusion

Emotional and behavioural difficulties among primary school pupils vary by school and this is not completely explained by pupil characteristics including socioeconomic status, ethnicity, and parental mental health. Interventions that address the school environment may be an effective way to improve the mental health of young people.

### Supplementary Information

Below is the link to the electronic supplementary material.

Supplementary file1 (PDF 383 KB)

## Data Availability

Researchers can access data by contacting the Born In Bradford team: https://borninbradford.nhs.uk/research/.
