# Supplementary material for: How do schools influence the emotional and behavioural health of their pupils? A multi-level analysis of 135 schools in the Born in Bradford inner city multi-ethnic birth cohort
Source: Soc Psychiatry Psychiatr Epidemiol. 2024 Jan 9;59(8):1335–46. doi: 10.1007/s00127-023-02608-8 (PMC11291525; doi:10.1007/s00127-023-02608-8)
Supplement: Supplementary file 1 — Supplementary file1 (PDF 383 KB) [file 127_2023_2608_MOESM1_ESM.pdf]

Supplementary information

1. Strengths and difficulties questionnaire items 2
2. Distribution of SDQ subscale scores.. 3
3. References for supplementary information.. 4

1. Strengths and difficulties questionnaire items

The Strengths and Difficulties Questionnaire comprises 25 items grouped into five subscales.[1] In this study, the questionnaire was completed by a parent. Each item is rated as ‘not true’, ‘somewhat true’, or ‘always true’. Some items are negative (ie. contribute to the score if somewhat or always true), and others are positive (ie. contribute to the score if not or somewhat true). Scoring is described elsewhere.[2]

Table 1: Strengths and Difficulties Questionnaire items

Item number	Subscale	Item
5	Conduct	Often has temper tantrums or hot tempers
7		Generally obedient, usually does what adults request
12		Often fights with other children or bullies them
18		Often lies or cheats
22		Steals from home, school or elsewhere
3	Emotional	Often complains of headaches, stomach-aches or sickness
8		Many worries, often seems worried
13		Often unhappy, down-hearted or tearful
16		Nervous or clingy in new situations, easily loses confidence
24		Many fears, easily scared
2	Hyperactivity	Restless, overactive, cannot stay still for long
10		Constantly fidgeting or squirming
15		Easily distracted, concentration wanders
21		Thinks things out before acting
25		Sees tasks through to the end, good attention span
6	Peer	Rather solitary, tends to play alone
11		Has at least one good friend
14		Generally liked by other children
19		Picked on or bullied by other children
23		Gets on better with adults than with other children
1	Prosocial*	Considerate of other people's feelings
4		Shares readily with other children (treats, toys, pencils etc.)
9		Helpful if someone is hurt, upset or feeling ill
17		Kind to younger children
20		Often volunteers to help others (parents, teachers, other children)

* Items from the prosocial subscale were not included in this research

2. Distribution of SDQ subscale scores

Figure 1: Distribution of SDQ subscale scores among 5,306 participants in the Born In Bradford questionnaire, at age 7-11

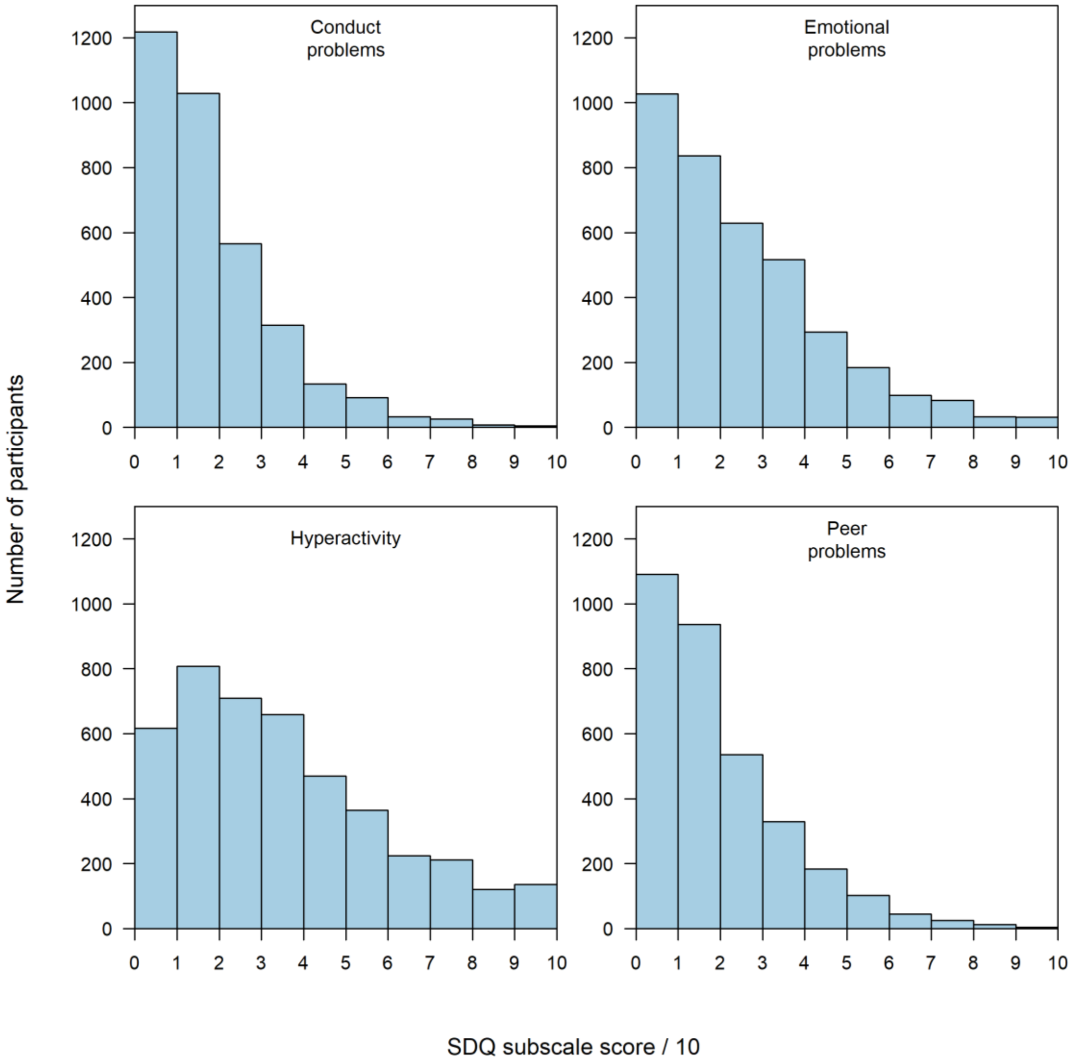

3. References for supplementary information

1 Goodman R. The Strengths and Difficulties Questionnaire: A Research Note. *J Child Psychol & Psychiat* 1997;**38**:581–6. doi:10.1111/j.1469-7610.1997.tb01545.x

2 Youth In Mind. The Strengths and Difficulties Questionnaire. https://www.sdqinfo.org/ (accessed 6 Jan 2023).
